# Antioxidant and antidiabetic profiles of two African medicinal plants: *Picralima nitida* (Apocynaceae) and *Sonchus oleraceus* (Asteraceae)

**DOI:** 10.1186/1472-6882-13-175

**Published:** 2013-07-15

**Authors:** Clautilde Mofor Teugwa, Pascaline Chouadeu Mejiato, Denis Zofou, Bruno Tugnoua Tchinda, Fabrice Fekam Boyom

**Affiliations:** 1 Laboratory of Phytobiochemistry and Medicinal Plants Studies, Department of Biochemistry, Faculty of Science, University of Yaoundé I, P.O. Box: 812, Yaoundé, Cameroon; 2 Laboratoire de Phytoprotection et de valorisation des resources végétales, Department of Biochemistry, Faculty of Science, University of Yaoundé I, Biotechnology Centre, Yaoundé, Cameroon; 3 Biotechnology Unit, University of Buea, Buea, South West Region, Cameroon

**Keywords:** Antioxidant, Diabetes, Oxidative stress, Hypoglycaemic activity, *Sonchus oleraceus*, *Pricralima nitida*

## Abstract

**Background:**

Diabetes mellitus (DM) is a metabolic disease characterized by chronic hyperglycaemia generally associated with oxidative stress. The present study aims at evaluating the antioxidant and antidiabetic potential of methanol and hydroethanol extracts of the stem bark and leaves of *Pricralima nitida* and the *Sonchus oleraceus* whole plant respectively.

**Methods:**

The *in vitro* antioxidant activity was assessed using 1,1-Diphenyl-2-picrilhydrazyl (DPPH) for free radical-scavenging properties of the extracts, and the Folin-Ciocalteu method in determining their phenol contents. The antidiabetic activity was tested in mice following streptozotocin diabetes induction, and selected oxidative stress markers (Malondialdehyde, Hydrogen peroxides and Catalase) were measured in order to evaluate the level of oxidative stress in treated animals.

**Results:**

The *in vitro* antioxidant activity using DPPH showed IC_50_ ranging from 0.19 ± 0.08 to 1.00 ± 0.06 mg/mL. The highest activity was obtained with the hydroethanol extracts of *S. oleraceus* (0.19 mg/mL and *P. nitida* (0.24 mg/mL). Polyphenol contents ranged from 182.25 ± 16.76 to 684.62 ± 46.66 μg Eq Cat/g. The methanol extract of *P. nitida* showed the highest activity, followed by the hydroethanol extract of *S. oleraceus* (616.89 ± 19.20 μEq Cat/g). The hydroethanol extract of whole plants (150 mg/Kg) and methanol leave extract of *P. nitida* (300 mg/Kg) exhibited significant antidiabetic activities with 39.40% and 38.48% glycaemia reduction, respectively. The measurement of stress markers in plasma, liver and kidney after administration of both extracts showed significant reduction in MDA and hydrogen peroxide levels, coupled with a substantial increase in catalase activity.

**Conclusions:**

These findings suggest that *S. oleraceus* whole plant and *P. nitida* leaves possess both antidiabetic and antioxidant properties, and therefore could be used as starting point for the development of herbal medicines and/or source of new drug molecules against diabetes.

## Background

Diabetes mellitus is a metabolic disease characterized by chronic hyperglycaemia and alteration of carbohydrate, proteins and lipids metabolism associated, with abnormald secretion and/or activity of insulin [1]. The impaired metabolism is often accompanied with excessive release of free radicals through lipid peroxidation, due to alteration of the activity of several proteins [2], aggravated by a drastic drop in antioxidant immune mechanisms [3]. This imbalance generally lead to oxidative stress which is the main factor associated with the severity and death in diabetes. Diabetes mellitus is a major cause of disability, figuring among the top ten killers worldwide [4]. The disease is rapidly spreading in Africa today, as a result of rapid and uncontrolled urbanization and westernization of lifestyle and dietary habits. Mbanya et al. [5] reported a prevalence varying widely across the continent: Benin 3%; Mauritania 6.0%; Cameroon 6.1%; Congo 7.1%; Zimbabwe 10.2%; Democratic Republic of Congo 14.5%. It is therefore a justified fear that DM with its accompanying generalized syndromes would become the next scourge in Africa, if a particular attention fails to be taken both in prevention of the upset and the treatment of the disease. Managing the growing number of disease cases is a permanent challenge, because most of the drugs in current use are seriously limited by both their side effects and the cost of the treatment. It is then an urgent need to search for new antidiabetic drugs which ideally should be not only more efficacious and less toxic than the current ones, but also cheap and available especially in rural areas. Previous ethnobotanical surveys revealed common use of plant materials in Africa for diabetes treatment [6]. *Sonchus oleraceus* and *Picralima nitida*, two multipurpose medicinal plants of Cameroonian pharmacopeia, are widely used to treat DM across the African continent [7-9]. In Cameroon in particular, the stem bark and leaves of *P. nitida*, and the whole plant of *S. oleraceus* are among the common phytomedicines used by traditional healers to treat diabetes [8,9]. Although the activity of the seed of *P nitida* has been established [8,9]; the major plant parts (stem bark and leaves) in common use are yet to be investigated for their acclaimed efficacies. This prompted us to initiate the present study which aims at evaluating the antioxidant and antidiabetic profiles of crude extracts prepared from these two plant species, following prescriptions of traditional healers.

## Methods

### Plant materials

Stem bark and leaves of *P. nitida* (Voucher number 45138/HNC) were collected in May 2009 in the Bakassa village (West Region, Cameroon) and the *S. oleraceus* (Voucher number 37069/HNC) whole plant harvested in the University of Yaoundé I Campus (Centre Region, Cameroon). The identification of both plants species were confirmed by the Cameroon National Herbarium in Yaoundé where Voucher specimens were deposited.

### Extract preparation

The different plant parts collected were air-dried in the dark and ground to powder. For the methanol extraction, 200 g each of *S. oleraceus* powder whole plant and *P. nitida* stem bark and leaves, were separately macerated in 500 mL of methanol for three days with thorough homogenization twice per day. Regarding the hydroethanol extraction, 500 mL of distilled water - 95% alcohol (1:1, v/v) was used to macerate 200 g each of powder from *S. oleraceus* and similarly as for methanol extraction. At the end, each macerate was filtered with Whatman No 1 filter paper, and the filtrate evaporated to dryness using a rotatory evaporator (Bűchi 011, USA).

### Phytochemical analysis

The extracts were screened for detection of different chemical families according to the methods previous described by Odebeji and Safowara [10]. In brief, phenolic compounds were detected using the ferrocyanide reaction; triterpenes and sterols were revealed by their reactivity with anhydrous acetate and sulphuric acid. Alkaloids were detected using sulphuric acid, whereas the presence of saponins revealed based on their foaming property. Tanins and flavonoids were revealed using ferric chloride and hydrochloric acid, respectively. Anthraquinones were detected in extracts by the chloroform/petroleum system, while the presence of lipids was assessed on filter paper.

### DPPH radical-scavenging activity

The stable 1,1-diphenyl-2-picryl hydrazyl radical (DPPH) was used for determination of free radical-scavenging activity of the extracts as described by Hatano et al. [11] with slight modification [12]. Different concentrations (0.25, 0.5, 1 and 2 mg/mL) of each extract were prepared in distilled water, 30 μL of each solution mixed with 1 mL of ethanol solution of DPPH (0.1 mM) and incubated for 30 min in the dark. At the end of this period, the absorbance was recorded at 517 nm using a spectrophotometer, and the antiradical activity of each concentration calculated as percentage reduction of DPPH concentration, with reference to the optical density at the start, as followed:

% scavenged [Free radicals] = [(A_o_ − A1)/A_o_] × 100 where A_o_ was the absorbance of the control and A_1_ was the absorbance in the presence of the sample of extract or standard.

The IC_50_ values were then generated by extrapolation from the curve of activity versus concentration.

### Reduction potential of the extracts

In order to investigate the reduction potential of the different extracts, their polyphenol contents were determined using the method described of Folin-Ciocalteu as described earlier by Singleton and Rossi [13], with some modifications [14]. In brief, 30 μL of extract of known concentration were thoroughly mixed with 10 mL of Folin-Ciocalteu (Sigma) 0.2 N and the absorbance measured at 750 nm, after 30 min incubation at room temperature in the dark. Catechine solutions in methanol at 10, 20, 30, 40 and 60 μM were used as standards.

### Diabetes induction in rats

The animals were kindly provided by the Animal House of the Department of Animal Biology and Physiology, Faculty of Science at the University of Yaoundé I (Cameroon). The antidiabetic profiles of the extracts with significant antiradical and antioxidant activities was assessed using two complementary approaches: the glucose intolerance test (hypoglycaemic activity), and the evaluation of activity in rats with induced diabetes.

### Hypoglycaemic activity

A total of 45 three month-old *Wistar* rats (20 males, 25 females) with body weight of 285-310 g, were divided into 9 groups with comparable average body weight. The different treatments were administered orally as indicated in Table 1. During the period of treatment, water was available to the mice *ad libitum*. Fasting blood sugar (FBS) was first measured for all the animals before administration of the different treatments. The different extracts and Glibenclamide (positive control) were then administered orally 30 min after administration of glucose. FBS was then taken 0.5, 1.5, 2.5, 3.5 and 4.5 hours post hyperglycaemia induction. Each time, FBS was measured from a blood drop collected from the rat’s tail, using a glucometer (Accucheck, USA). The hypoglycaemic potential of the extracts was evaluated as their ability to correct the hyperglycaemia 4.5 hours after induction.

**Table 1:** Treatment of different groups of mice for the hypoglycaemic activity assay

**Group**	**Treatment**
1	Distilled water (5 mL/Kg)
2	2 g/Kg glucose + Distilled water (5 mL/Kg)
3	2 g/Kg glucose + 10 mg/Kg Glibenclamide (Positive control)
4	2 g/Kg glucose +75 mg/Kg PNLM
5	2 g/Kg glucose +150 mg/Kg PNLM
6	2 g/Kg glucose +300 mg/Kg PNLM
7	2 g/Kg glucose +75 mg/Kg SOWH
8	2 g/Kg glucose +150 mg/Kg SOWH
9	2 g/Kg glucose +300 mg/Kg SOWH

**PNLM:** Methanol extract of *Picralima nitida* leaves; **SOWH:** Hydroethanol extract of *Sonchus oleraceus* whole plant.

### Antidiabetic activity

The acute and sub-acute activities of the different extracts were determined in rats with streptozotocin-induced diabetes, following the method of Al-Shamaony et al. [15]. Male rats were subjected to overnight fasting prior to the diabetes induction. Each of them subsequently received an intravenous injection of 50 mg/Kg streptozotocin (Sigma) dissolved in 0.1 citrate buffer at pH 4.5. After 3 days, the animals with at least 250 mg/dL FBS were considered “diabetic” and used for the assays.

### Acute activity

Seven groups of 5 rats each were based on their body weight, and treated as shown by Table 2. FBS was measured immediately before drug administration (0 h), and subsequently 1, 3 and 5 hours after drug administration, with all the animals maintained fasting and receiving water ad libitum. The acute antidiabetic potential of the extracts was determined as the ability of the single dose treatment to drop FBS in 5 hours.

**Table 2:** Treatment of different groups of mice for the antidiabetic activity assay

**Group**	**Treatment**
1	Distilled water (5 mL/Kg)
2	2 g/Kg glucose + Distilled water (5 mL/Kg)
3	2 g/Kg glucose + 10 mg/Kg Glibenclamide (Positive control)
4	2 g/Kg glucose +150 mg/Kg PNLM
5	2 g/Kg glucose +300 mg/Kg PNLM
6	2 g/Kg glucose +150 mg/Kg PNLM
7	2 g/Kg glucose +300 mg/Kg SOWH

**PNLM:** Methanol extract of *Picralima nitida* leaves; **SOWH:** Hydroethanol extract of *Sonchus oleraceus* whole plant.

### Sub-acute activity

A total of 35 rats were divided into 7 groups of 5 each and treated similarly as for the acute activity study. But in this case, the different drugs were administered daily, for 14 consecutive days, and FBS measured on days 0 (before drug administration), 4, 8 and 14 (end of the assay). At the end of the assay, the rats were sacrificed, blood collected in heparin to prepare plasma. The liver and kidneys were equally extracted and ground to form homogenates. The plasma and organ homogenates prepared were stored at -20°C until required for measurement of the markers of oxidative stress (Malondialdehyde, Catalase activity and hydrogen peroxide).

### Titration of markers of oxidative stress in treated rats

#### Malondialdehyde

Serum MDA levels were estimated by the method of Yagi [16] using thiobarbituric acid (TBA). According to this method, the acid reacts with MDA to form a stable pink colour with maximum absorption at 535 nm. The reagent was prepared by mixing 375 mg of thiobarbituric acid, 20 g of Trichloroacetic acid, 10 mg of butyl-hydroxytoluene, 20 mL of HCl 1 N, in 50 mL of distilled water. The mixture was heated at 40°C until total dissolution of crystals, and the volume filled up to 100 mL with distilled water. 400 μL of this reagent was mixed with 300 μL of homogenate (10% w/v) and tightly capped in a glass tube protected from light with aluminium foil. The mixture was then boiled in a water bath at 100°C for 15 min, and the tubes allowed getting cold for about 30 min on ice. The mixture was subsequently centrifuged at 3000 rpm for 5 min and the absorbance of the supernatant recorded at 532 nm. The MDA content of the sample was calculated as follow:

$$\mathrm{MDA}\;\left(\mathrm{mol}/L\right)=\left[A_{o}–A_{1}\right]/\epsilon \;x\;l\;x\;V$$

Where A_1_ was the absorbance of the test sample; A_o,_ absorbance of the negative control; ℇ = 1.53.105 M^-1^/Cm; l the diameter of the cuvette (l = 1 cm); and V was the total volume of the supernatant collected.

#### Hydrogen peroxide

The hydrogen peroxide content was determined by a methods previously described by Jiang et al. [17]. Briefly, the (FOX) ferrous oxidation in xylenol orange) reagent was prepared by mixing 88 mg of butylated hydroxytoluene, 7.6 mg of xylenol orange, 9.8 mg of ammonium sulphate, 90 mL of methanol and 10 mL of sulphuric acid, 250 mM. The mixture was then thoroughly homogenized before used. In a test tube, 100 μL of sample or distilled water (blank) was added to 900 μL of FOX reagent, homogenized and incubated at 37°C for 30 min. At the end, the absorbance was recorded at 560 nm and the hydrogen peroxide concentration was determined as followed:

$$\left[H_{2}O_{2}\right]\;\left(\mathrm{\mu mol}/L\right)=\left[A_{o}–A_{1}\right]/\epsilon \;x\;l\;x\;V$$

Where A_1_ was the absorbance of the test sample; A_o,_ the absorbance of the blank; ℇ = 1.53.105 M^-1^/Cm; l the diameter of the cuvette (l = 1 cm), and V was the total volume of the mixture.

#### Catalase

The catalase activity in blood and organ homogenate was measured by the method of Sinha [18]. For each of the tubes in a series of 3 per sample, 100 μL of the sample or distilled water (blank) was added to 250 μL of 1 time phosphate buffer and 200 μL of H_2_O_2_, and the reaction stopped after 0, 30 and 60 seconds for tube 1, 2, and 3 respectively. The reaction was stopped by adding 1 mL of dichromate-acetic acid mixture (100 mL of potassium dichromate dissolved in 300 mL of acetic acid). The tubes were then boiled at 100°C for 10 min, allowed to get cold and the absorbance recorded at 620 nm, for each incubation time. The OD values obtained were plotted against time in Microsoft Excel 2010 and the catalase activity calculated.

### Statistical analysis

Each test was conducted at least in triplicate and all the replicate values pooled together into Mean ± Standard variation. The different test groups were compared to each other using ANOVA, whereas variations within the same group were evaluated using pair-*t* test. Correlations between the different activities were assessed. All the statistical analysis was conducted in SPSS at 95% and 99% confidence intervals.

## Results

### Phytochemical profiles of the different extracts

The methanol extract of the leaves of *P. nitida* scored the highest extraction yield (28.17%), followed by the hydroethanol and methanol extracts of *S. oleraceus* (19.11% and 13.02% respectively), whereas methanol extraction of *P. nitida* stem bark had the lowest yield (5.39%). The phytochemical analysis of the different extracts revealed the presence of a diversity of chemical families, including flavonoids, terpenes, sterols, saponins, alkaloids and polyphenols. However, anthocyanines and lipids were absent in all the extracts.

### Free radical-scavenging and reduction potentials of the extracts

**Figure 1:**
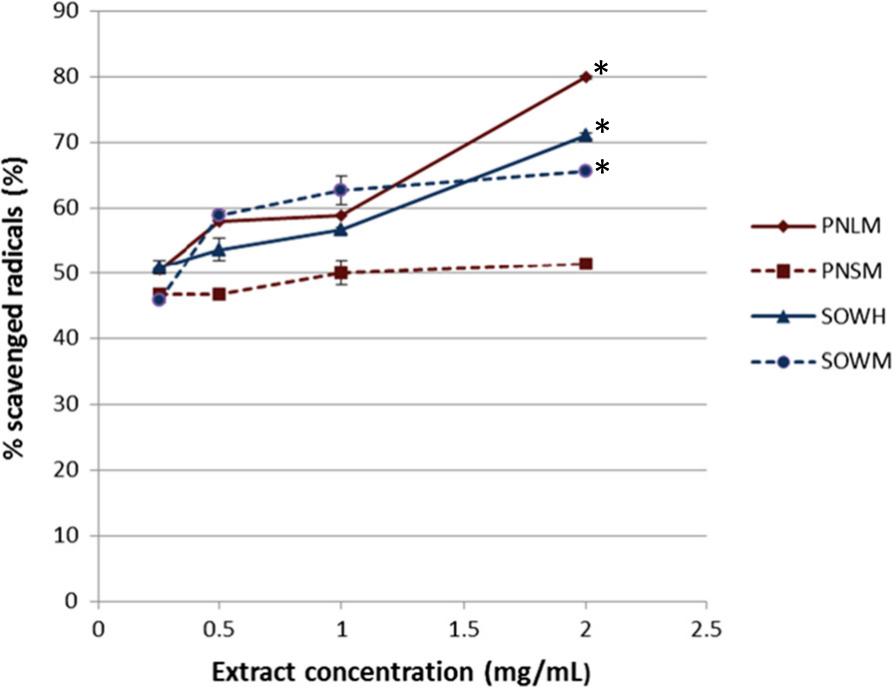
**DPPH free radical scavenging potential of *P. nitida *and *S. oleraceus *extracts.** PNLH Methanol extract of *P. nitida* leaves; PNSM: Methanol extract of *P. nitida* stem bark; SOWH: Hydroethanol extract of *S.oleraceus* whole plant; SOWM: Methanol extract of *S. oleraceus* whole plant. *% significantly higher than the one at 0.25 mg/mL at 99% confidence interval.

Figure 1 presents the free radical-scavenging potential of the different extracts against DPPH. It clearly appears a significant increase in this potential with concentration for the methanol extracts of *P. nitida* leaves and the hydroethanol extract of *S. oleraceus* whole plant (p < 0.01); unlike the *P*. *nitida* stem bark and the methanol extract of *S. oleraceus* which did not show any significant change in activity with the extract concentration. Based on their IC_50_s, the hydroethanol extract of *S. oleraceus* exhibited the highest activity with a value of 0.19 ± 0.08 mg/mL which is very close to the positive control catechine (IC_50_ = 0.13 ± 0.00 mg/mL). The methanol extract of *P. nitida* leaves and the hydroethanol extract of *S. oleraceus* showed moderate activities with IC_50_s of 0.24 ± 0.03 and 0.28 ± 0.04 mg/mL, respectively, whereas the stem bark of *P. nitida* had the lowest activity (IC_50_ = 1 mg/mL).

Regarding polyphenol content, methanol extract of the *P. nitida* leaves showed the highest concentration (684.62 ± 46.66 μg Eq cat/g of dry matter), followed by the hydroethanol extract of *S. oleraceus* (616.89 ± 19.20 μg Eq cat/g) whereas the methanol extract of the stem bark of *P. nitida* and whole plant of *S. oleraceus* had the lowest polyphenol concentrations (215.31 ± 15.10 and 182.25 ± 16.76 μg Eq cat/g, respectively).

Based on their high polyphenol contents, combined with the significant free radical-scavenging activities, and their phytochemical profiles, the methanol extract of *P. nitida* leaves and the hydroethanol extract of *S. oleraceus* were considered for *in vivo* antidiabetic studies.

### Hypoglycaemic activity

**Figure 2:**
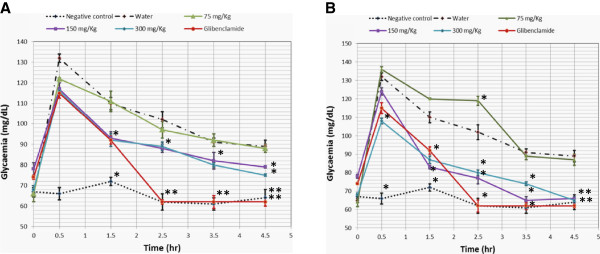
**Hypoglycaemic activities of the extracts of *P. nitida *and *S. oleraceus *extracts.** The values presented are in the form of mean and standard deviation of 5 different measurements. **A**: Activity of *S. oleraceus* extracts and the controls; **B**: Activities of *P. nitida* extracts and the controls. * Value statistically different from the corresponding value of the control (water) at 99% Confidence Interval. ** Value highly different from the corresponding value of the control (water) at 99% Confidence Interval.

The variations of glycaemia following oral glucose administration, in the presence of the different extract and in controls are summarized in Figure 2. There was a significant rise in glycaemia in all the groups 30 min after glucose administration, but more pronounced in the untreated group and in the one receiving 75 mg/kg of *P. nitida* extract. As from 1 hr post-induction, a significant drop was observed with all the extracts, with a higher activity with *S. oleraceus* extract at 150 and 300 mg/kg and Glibenclamide (p < 0.01). The dose of 75 mg/Kg therefore did not appear to have any significant effect on the glycaemia in postprandial rats. The doses of 150 and 300 mg/Kg were then retained for subsequent work. With regards to the *P. nitida* extract, there was a continuous decrease in glycaemia in the animals receiving 150 and 300 mg/Kg, but no significant effect observed with 75 mg/Kg. This lower dose was therefore left out in subsequent studies, like in the case of *S. oleraceus*.

### Acute antidiabetic activity

A dose-dependent and time-dependent drop in glycaemia was observed with the *P. nitida* extract and the positive control (p < 0.05). The 300 mg/Kg dose showed 34.75% reduction, compared with 22.40% obtained with 150 mg/Kg, and 39.78% for Glibenclamide. The glycaemia remained statistically constant all through the experiment, in both the untreated rats and those receiving distilled water. A significant reduction in glycaemia was observed in all treated groups, but not dose-dependent: 35.89% for 150 mg/Kg against 16.96% for 300 mg/Kg.

### Sub-acute antidiabetic activity

**Figure 3:**
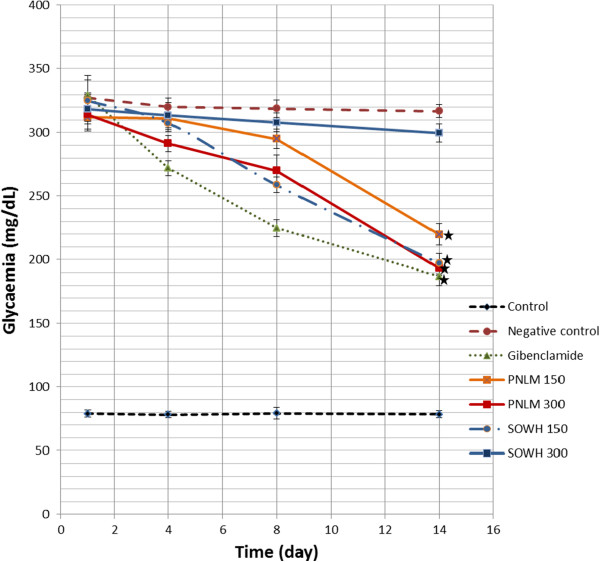
**Sub-acute antidiabetic effects of *P. nitida *and *S. oleraceus *extracts in postprandial rats.** Each value is presented as mean and Standard deviation of a 5 different measurements. * : Value statistically different from each of the controls at 95% Confidence Interval.

Like in acute activity, glycaemia remained unchanged in untreated rats as well as in those under distilled water during entire period of the experiment (Figure 3). Glibenclamide used as positive control exhibited the highest activity with a final decrease of 43.21% on day 14, followed by *S. oleraceus* at 150 mg/kg (39.40%), *P. nitida* at 300 mg/kg (38.48%) 150 mg/kg (29.62%), while *S. oleraceus* at 300 mg/kg instead showed a very weak activity, with 5.91% reduction only.

### Effects of the extracts on the markers of oxidative stress

**Figure 4:**
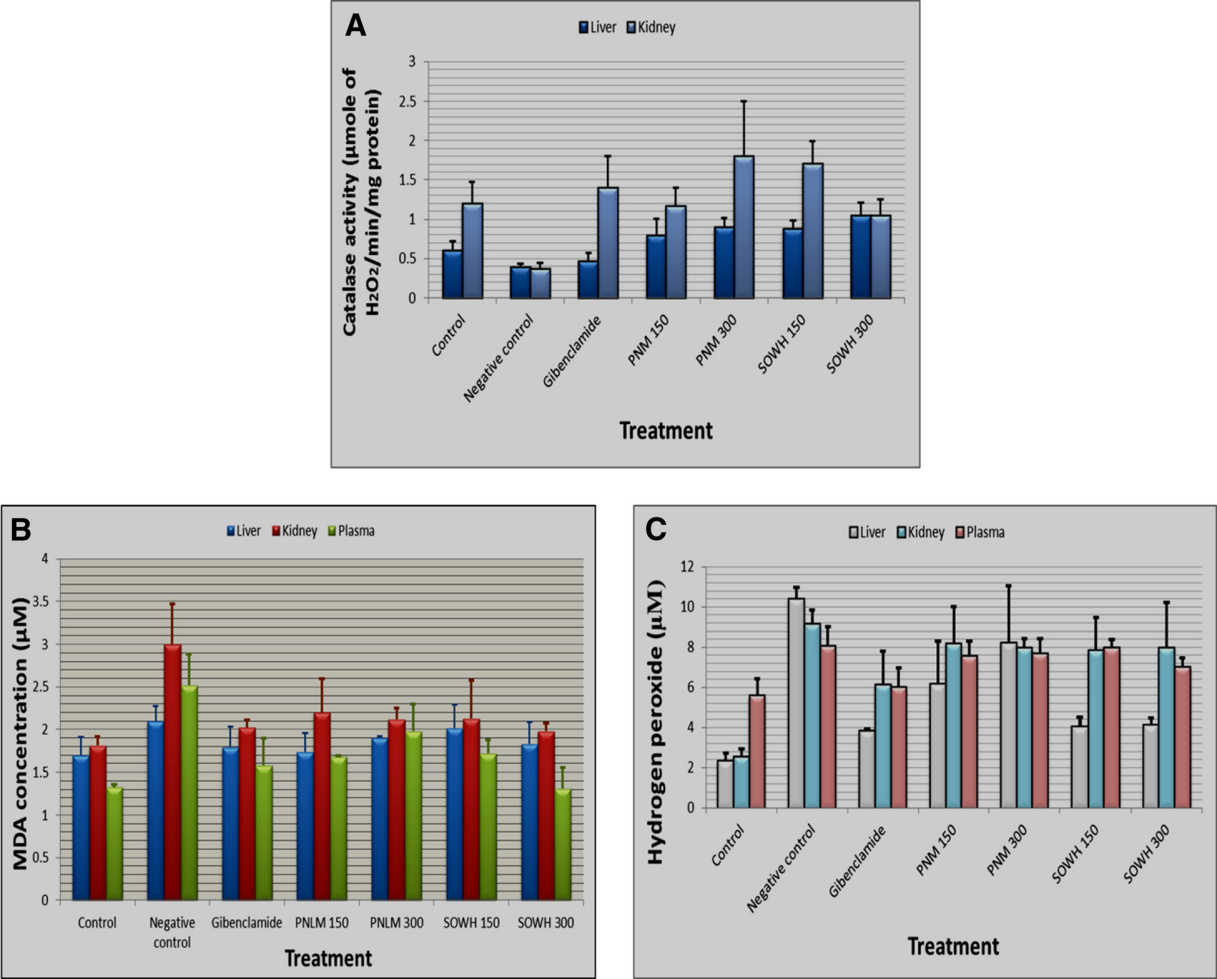
**Effects of the different extracts on oxidative stress markers in diabetic rats. A**: Effect on catalase; **B**: effect on MDA; **C**: Effect on hydrogen peroxide. Results are presented as mean and standards deviation from five measurements for each parameter.

Figure 4 summarizes the effects of the different extract concentrations on the concentrations of MDA, hydrogen peroxide and catalase in liver, kidney and plasma. In general, the MDA concentration (Figure 4B) was significantly higher in the liver, kidney and plasma of the untreated rats, compared with those receiving extracts or Glibenclamide. The reductions were comparable for all the treatment and doses, except for *S. oleraceus* at 150 mg/Kg where the extract was ineffective in reducing the MDA production in the liver, though active in the kidney and plasma. In almost all the groups and considering all the three organs, MDA values were slight higher than in non-diabetic animals. Concerning hydrogen peroxide (Figure 4C), generally, the concentrations in treated rats were significantly lower than the values for the untreated (p < 0.05); with *S. oleraceus* at 150 mg/kg as the most active. The effects were not dose-dependent in both *P. nitida* and *S. oleraceus* extracts. The catalase activity (Figure 4A) significantly increased in rats receiving either the plant extracts or the reference drug) as compared with untreated animals (p < 0.05). This catalase stimulating effect was even more pronounced in the liver (0.79 – 1.05 μmol H_2_O_2_/min/mg of protein) than in the positive control Glibenclamide (0.47 ± 0.10 μmol H_2_O_2_/min/mg). In the kidney, *P. nitida* at 300 mg/kg had the highest catalase activity (1.80 ± 0.70 μmol H_2_O_2_/min/mg) followed by *S. oleraceus* at 150 mg/kg (1.70 ± 0.29 μmol H_2_O_2_/min/mg), still being more active than the reference drug (1.40 ± 0.40 μmol H_2_O_2_/min/mg). The activity was dose-dependent for *P. nitida*, but not for *S. oleraceus.*

## Discussion

The assessment of the antioxidant and antidiabetic potential of *P. nitida* and *S. oleraceus,* was achieved in the present study, through determination of 1) the phytochemical profiles and polyphenol contents of the extracts, 2) their free radical-scavenging activity, 3) the hypoglycaemic activity in postprandial rats, their acute and sub-acute antidiabetic activity in streptozotocin-induced rats, and 4) the ability of the extracts to stimulate fight against oxidative stress in diabetic rats. The phytochemical screening revealed the presence of flavonoids, terpenes, sterols, saponins, alkaloids and polyphenols which are potential bioactive molecules of these plants [19]. Stanley *et al*. [20] earlier reported the antidiabetic activity of glycosides, saponins, alkaloids, flavonoids and polyphenols. The methanol extract of *P. nitida* leaves and the hydroethanol extract of *S. oleraceus* whole plant showed higher polyphenol contents, free radical-scavenging activity, significant blood sugar reduction capacity, and reduced the levels of oxidative stress markers (MDA, H_2_O_2_ and catalase) pointedly. There was a significant correlation between the polyphenol content and both oxidative and hypoglycaemic activity, suggesting polyphenols as the main determinant of antioxidant and hypoglycaemic effects. Phenolic compounds are thought to be produced by plants to protect them from abiotic and biotic stresses, but they are also beneficial to humans under disease induced oxidative stress [21]. Afolabi et al. [22] also confirmed the correlation between polyphenol content and free radical-scavenging activity of plant extracts. Based on their high polyphenol contents, combined with the significant free radical-scavenging activities, and their phytochemical profiles, the methanol extract of *P. nitida* leaves and the hydroethanol extract of *S. oleraceus* were considered for *in vivo* antidiabetic studies. The capacity of the extract to stimulate glucose storage in case of sudden hyperglycaemia was assessed in rats submitted to induced-hyperglycaemia, through ingestion of considerable amount of sugar. Efficient doses in both extracts were 150 mg/Kg and 300 mg/Kg. The drop in blood sugar observed may be due to either a stimulation of insulin secretion and activity, or other factors. Again, polyphenols and other metabolites present in these extracts (sterols, alkaloids, and flavonoids) were previously shown to have hypoglycaemic activity [20,23,24].

In acute and sub-acute studies, significant effects were observed as from 3 h. On day 14, all the extracts showed good activity, except for *S. oleraceus* at 300 mg/Kg. In general, the hydroethanol extract of *S. oleraceus* exhibited the highest activity, reaching 39.40% reduction in blood sugar at 150 mg/Kg, compared to 38.48% for the methanol leave extract of *P. nitida* at 300 mg/Kg. However, a much surprising finding noticed was that this activity of *S. oleraceus* was not dose-dependent, the reduction being more pronounced at the lower dose (150 mg/Kg). Furthermore, no sign of toxicity was observed both in the animal behaviour and at organ level. Further investigation may be needed to clarify this situation, by testing the extract using a different animal model for example.

The oxidative stress has been associated with diabetic states in humans and streptozotocin (STZ)-induced diabetic rats [24-27]. During diabetes, chronic high blood sugar increases the production of reactive oxygen species (ROS) through glucose autoxidation [28]. Free radicals act by lipid peroxidation, releasing MDA in large amount. MDA content in liver, kidney and plasma can therefore inform on the level of cell damage and apoptosis in diabetic patients or animals [29]. Together with hydrogen peroxide and catalase, MDA is commonly used as oxidative stress markers in diabetes. Low contents in MDA and H2O2, combined with significant increase in catalase activity were observed in all rats treated with the extracts, confirming their antioxidant activity in streptozotocin-induced rats. These extracts may therefore significantly stimulate the elimination of free radical occurring with the incidence of diabetes.

The antioxidant and antidiabetic potential of other plant parts of *P. nitida* and *S. oleraceus* were previously studied using different solvent systems. Working on the seeds, Okonta and Aguwa [9] observed a significant drop in blood glucose with the glycosides extract, with reduction in fasting blood glucose levels of 38.6% (250 mg/Kg) and 22.9% (500 mg/Kg) in normoglycaemic, and 64.4% (250 mg/Kg) and 39.0% (500 mg/Kg) in hyperglycemic rats. Based on these findings, the authors hypothesize that the hypoglycaemic activity observed may be due to the presence of glycosides in the *P. nitida* seeds. However, in the present work, there was no glycoside in the leaves or stem bark extracts used. This observation highlights the significant difference between the phytochemical composition of the leaves and seeds of *P. nitida*. Polyphenol, alkaloids and sterols were noticed as the main metabolites of the leaves, whereas glycosides were shown to be predominant in the seeds [9]. The hypoglycaemic effect of *P. nitida* seeds was also confirmed by Nwakile and Okore [30].

Concerning previous work on *S. oleraceus*, a treatment of diabetic rats with the whole plant infusions was shown to produce a marked amelioration of the impaired glucose tolerance at all examined periods after oral glucose loading and the lowered insulin and C-peptide levels, improvement in serum lipid profile including decrease in serum total lipid, total cholesterol, triglyceride, LDL-cholesterol and vLDL-cholesterol levels and increase in HDL-cholesterol level [31]. The antioxidant defense system was equally investigated by these authors, showing hepatic lipid peroxidation profoundly decreased and the total thiol and glutathione concentrations with detectable increase. The present study using different extraction processes therefore corroborates the findings from this previous investigation.

In summary, the antioxidant and anti-diabetic activities observed with these two medicinal plants could be explained by: 1) the presence of free radical scavenging ingredients fighting oxidative stress, and the inhibitory effects of phenolic compounds exerted against glucose generating pathways (see Additional file 1).

## Conclusions

In conclusion, the present work showed the antioxidant and antidiabetic potential of the methanol extract of *P. nitida* leaves and the hydroethanol extract of *S. oleraceus* whole plant. Considering their polyphenol high content, their significant free radical-scavenging, hypoglycaemic activity and their ability to prevent oxidative stress in diabetic rats; further investigations on these two plants of Cameroon pharmacopoeia, is highly encouraged.

### Ethical considerations

Animals were handled according to the ethical guidelines of the Cameroon National Veterinary Laboratory (LANVET, Ministry of Livestock, Fisheries and Animal Industry) and the ARRIVE (Animal Research: Reporting *In Vivo* Experiments) guidelines.

## Competing interests

The authors declare that they have no competing interests.

## Authors’ contributions

CMT conceived the project and supervised the work all through, PCM collected the plants parts and took part in biological tests, DZ participated in work design and drafted the manuscript, BTT participated in biological tests, FFB took part of work design and biological tests. All the authors proofread and approved the manuscript before submission.

## Pre-publication history

The pre-publication history for this paper can be accessed here:

http://www.biomedcentral.com/1472-6882/13/175/prepub

## Supplementary Material

Additional file 1 1. Plant extracts; 2. Polyphenols and other secondary metabolites; 3. Free radical scavenging molecules; 4. Free radicals. Click here for file
