# Supplementary material for: Antioxidant and antidiabetic profiles of two African medicinal plants: Picralima nitida (Apocynaceae) and Sonchus oleraceus (Asteraceae)
Source: BMC Complement Altern Med. 2013 Jul 15;13:175. doi: 10.1186/1472-6882-13-175 (PMC3718716; doi:10.1186/1472-6882-13-175)
Supplement: Additional file 1 — 1. Plant extracts; 2. Polyphenols and other secondary metabolites; 3. Free radical scavenging molecules; 4. Free radicals. [file 1472-6882-13-175-S1.docx]

**1**

***Picralima nitida (leaves)***

***Sonchus oleraceus (whole plant)***

**Polyphenols**

**& other secondary metabolites**

**Free radical scavenging activity**

**Free radicals**


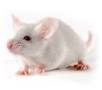


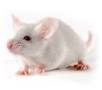


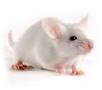


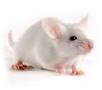


**Streptozotocin**

***Mouse with acute or sub-acute diabetes & oxidative stress***

***Healthy mouse:*** *Low blood sugar, reduced levels of MDA, H_2_O_2_ high level of Catalase*

***Mouse with low level of glucose***

***Hyperglycaemic mouse***

**Glucose**

**41**

**31**

**21**
